# Genomic characterization of the antiviral arsenal of Actinobacteria

**DOI:** 10.1099/mic.0.001374

**Published:** 2023-08-02

**Authors:** Héloïse Georjon, Florian Tesson, Helena Shomar, Aude Bernheim

**Affiliations:** ^1^​ Molecular Diversity of Microbes Lab, Institut Pasteur, Université Paris Cité, Inserm U1284, Paris, France; ^2^​ UMR 1137, IAME, Université de Paris, INSERM, Paris, France

**Keywords:** Actinobacteria, bacteriophages, defense systems, evolutionary genomics, *Streptomyces*

## Abstract

Phages are ubiquitous in nature, and bacteria with very different genomics, metabolisms, and lifestyles are subjected to their predation. Yet, the defence systems that allow bacteria to resist their phages have rarely been explored experimentally outside a very limited number of model organisms. Actinobacteria (Actinomycetota) are a phylum of GC-rich Gram-positive bacteria, which often produce an important diversity of secondary metabolites. Despite being ubiquitous in a wide range of environments, from soil to fresh and sea water but also the gut microbiome, relatively little is known about the anti-phage arsenal of Actinobacteria. In this work, we used DefenseFinder to systematically detect 131 anti-phage defence systems in 22803 fully sequenced prokaryotic genomes, among which are 2253 Actinobacteria of more than 700 species. We show that, like other bacteria, Actinobacteria encode many diverse anti-phage systems that are often encoded on mobile genetic elements. We further demonstrate that most detected defence systems are absent or rarer in Actinobacteria than in other bacteria, while a few rare systems are enriched (notably gp29-gp30 and Wadjet). We characterize the spatial distribution of anti-phage systems on *Streptomyces* chromosomes and show that some defence systems (e.g. RM systems) tend to be encoded in the core region, while others (e.g. Lamassu and Wadjet) are enriched towards the extremities. Overall, our results suggest that Actinobacteria might be a source of novel anti-phage systems and provide clues to characterize mechanistic aspects of known anti-phage systems.

## Introduction

The constant threat of viruses has led bacteria to develop a myriad of anti-phage systems. An impressive diversity of anti-phage molecular mechanisms has been characterized in the last 5 years, but still very little is known about their ecological roles in natural microbial communities. To date, anti-phage defence systems have been mostly studied in a few model organisms, notably *Escherichia coli* or closely related organisms [1–4]. Still, other types of bacteria can harbour very different genomics, metabolisms, and lifestyles. These changing evolutionary pressures could impact the types of defence systems they encode and/or their ecological implications.

Actinobacteria (Actinomycetota) are ubiquitous Gram-positive bacteria, which often have high G+C content. Several unique traits distinguish this phylum. First, many Actinobacteria have a complex life cycle, and can for instance form mycelia, sporulate, or undergo morphological differentiation [5–7]. Several actinobacterial genera also have genus-specific traits. For instance, mycobacteria have a unique cell wall, which might impact phage adsorption mechanisms [8–10]. Additionally, members of the genera *Streptomyces* and *Nocardia* have a remarkably rich secondary metabolism and encode for highly diverse reservoirs of bioactive compounds, making them a major source of clinically relevant drugs [11]. Finally, while most bacterial chromosomes are circular, Actinobacteria comprise several species with linear chromosomes, and notably members of the *Streptomyces* genus [12]. The linearity of their chromosome strongly impacts the genomic structure and organization of *Streptomyces* species. Notably, their chromosome is compartmentalized between a central region, which encompasses most of the core genes of *Streptomyces* and is flanked by two chromosomal arms encoding genes that are generally less conserved [13, 14].

Despite being a very diverse and widespread bacterial phyla of great economic importance, relatively little is known about the way Actinobacteria defend themselves against phages [15]. Yet, the unique characteristics of certain Actinobacteria might influence their anti-phage arsenal. For instance, the susceptibility to phage infection of actinobacterial species can vary depending on diverse development stages and cell forms [16]. It was also recently discovered that among the bioactive compounds produced by Actinobacteria, some display anti-phage activity [17, 18], suggesting links between their biosynthetic ability and the defence systems they encode. Finally, the distribution of anti-phage arsenals carried by linear chromosomes has not been described yet.

To determine if the unique characteristics of Actinobacteria confer to their anti-phage defence phylum-specific characteristics, we aimed to characterize both qualitatively and quantitatively the anti-phage arsenal of Actinobacteria and compared it to current knowledge in other common bacterial phyla.

## Methods

### Availability

A list of all genomes used in this analysis and corresponding phylogenetic information is available in (Table S1, available in the online version of this article). All defence systems and BGCs detected respectively by DefenseFinder [19] and antiSMASH [20] are available in Tables S2 and S3. The results of prophage detection by Virsorter [21] are available in Table S4. Data and code used for this analysis are available online at https://github.com/mdmparis/Actinobacteria_defense_systems.

### Figures and statistical analysis

Data analyses were done using pandas v 1.3.4 [22]. Figures were made with python, using modules matplotlib v3.4.3 3 [23], seaborn [24], and pycirclize. Statistics were computed using python modules scipy.stats [25], statsmodels [26] and statannotations.Annotator.

### Genome database

A total of 22  803 fully sequenced prokaryotic genomes were downloaded in July 2022 from the RefSeq database [27]. Among them are 2253 genomes of Actinobacteria, accounting for 207 actinobacterial genera and 790 actinobacterial species. All accession numbers and phylogenetic information are available in Table S1.

In addition, we curated a version of this database to remove the redundancy between genomes (see Discussion). Using the ‘prepare’ module of the PanACoTA software, we removed 333 genomes (among which 141 *Mycobacterium *, 88 *Corynebacterium,* 28 *Bifidobacterium *, and 27 *Streptomyces *), keeping a minimum Mash genetic distance between two genomes of 1e-4.

### Detection of defence systems

DefenseFinder v1.0.9 (with DefenseFinder models v1.2.2) [19] was used with default parameters to detect anti-phage genes and resulting anti-phage systems in the genomes of the RefSeq database [19]. This resulted in the detection of 380 556 anti-phage genes, accounting for 161 234 systems. Among these systems, 13 833 were from Actinobacteria. All results of DefenseFinder detection are available in Table S2.

### Detection of BGCs

All actinobacterial BGCs were detected by running antiSMASH v.6.0 [20] on all genomes of Actinobacteria of the RefSeq database. In 2253 genomes, 39 164 BGCs were detected. Different types of BGCs were readily annotated by antiSMASH.

### Characterization of the differential abundance of defence systems in Actinobacteria

To determine which anti-phage systems had a differential abundance in Actinobacteria compared to non-Actinobacteria, we built for each system an estimator designed to evaluate if the system is rarer or more abundant in this phylum compared to other bacteria. To do so, we calculated for each type of defence system the difference between its abundance (i.e. the average number of this system encoded in one genome) in Actinobacteria and its abundance in non-Actinobacteria. Contrary to frequency, the abundance measure takes into account the fact that some genomes encode several copies of the same system. Because the abundances of defence systems are very heterogeneous, we normalized this difference by the general abundance of the system. By doing so, we obtained the following indicator:

$$Estimator_{system}\;=\frac{Abundance_{system}\left(Actinobacteria\right)-\;Abundance_{system}\;\left(non\;Actinobacteria\right)}{Abundance_{system}\left(All\;bacteria\right)}$$

**Fig. 1.**
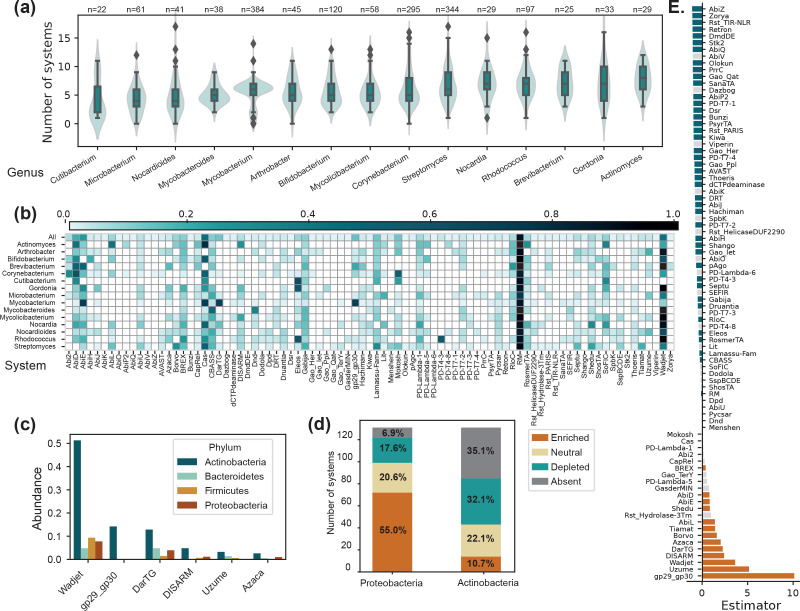
Distribution of defence systems in Actinobateria. (**a.)** Number of defence systems encoded per genome depending on genera of Actinobacteria. The x-axis is cut at *n*=19. (**b.)** Proportion of the genomes of genera of Actinobacteria that encode different types of defence systems. In (a.) and (b.), only genera containing more than 20 fully sequenced genomes were represented. (**c.)** Frequency in major bacterial phyla of the six types of systems the most enriched in Actinobacteria. (**d.)** Proportion of defence systems that are absent, enriched or depleted compared to other bacteria in Actinobacteria versus in Proteobacteria. (**e.)** Estimators (see formula in Methods) of the differential abundance of different types of actinobacterial systems compared to non-Actinobacteria. Coloured bars (orange: enriched, blue : depleted) represent a significant difference of the abundance of a system in Actinobacteria compared to non-Actinobacteria (*P*≤0.05, ANOVA corrected by Bonferroni).

Thus, a negative indicator indicates a system rarer in Actinobacteria than in other bacteria and a positive one indicates a system more abundant in Actinobacteria than in other bacteria (Fig. 1c). ANOVA tests corrected by Bonferroni were used to evaluate the difference between the mean number of occurrences of each type of systems per genome encoded by the two groups.

### Building of the phylogenetic trees

For each of the major actinobacterial genera, a core-genome-based phylogenetic tree was generated using PanACoTA v. 1.3.1 [28]. For each of the genera, we took all the chromosomes (and not plasmids) of the genomes it comprises. For each genus, between one and five chromosomes of closely related genomes were selected as outgroups. We then computed the pan genome of this genus using the PanACotA pangenome module with default parameters (pangenome min identity : 80 %), and computed the core genome using the corepers module, with -t 0.95 (meaning that to be considered persistent families need to contain one member in at least 95 % of the genomes). Alignments of the core genomes were generated using PanACotA align (default parameters). Finally, the tree module of PanACota was used to generate the corresponding phylogenetic trees using IQtree (default option) with a bootstrap value of 1000.

Phylogenetic trees were displayed and annotated using iTOL [29].

### Measure of phylogenetic signal on defence systems distribution

Phylogenetic signals were measured for each anti-phage system encoded by *Rhodococcus* and *Streptomyces* were measured using Pagel’s lambda. Both Pagel’s lambda and corresponding *p*-values were measured using the phylosig function from the phytools R package [30].

### Identification of MGE-encoded anti-phage systems and BGCs

The RefSeq annotation of replicon types was used to distinguish plasmids from chromosomes. Among actinobacterial replicons, 2255 were annotated as chromosomes and 878 as plasmids. Virsorter v.2.2.3 (min length set to 2000) was then used to detect prophages in all actinobacterial replicons [21]. Out of 2253 Actinobacteria genomes, 1656 encoded at least one prophage. The results of Virsorter’s detection are available in Table S4.

Due to the possibility of an imperfect detection of prophage boundaries by Virsorter, all defence systems and BGCs that were at least partially included within the boundaries of a given prophage were considered as prophage-encoded. More precisely, multigene anti-phage systems were considered prophage encoded if at least one of their genes was encoded within a prophage, while BGC regions were considered prophage encoded if at least 50 % of the region was contained within a prophage.

### Characterization of the spatial distribution of BGCs and defence systems in *Streptomyces*

For the characterization of the distribution of defence systems and BGCS on *Streptomyces* linear replicons, only chromosomes or plasmids annotated as linear were selected. Out of 344 *Streptomyces* chromosomes, 12 were annotated as circular. Although this is most likely misannotations, these chromosomes were excluded from our analysis. Out of 183 *Streptomyces* plasmids, 120 were annotated as linear. As *Streptomyces* are known to encode both linear and circular plasmids, only these 120 plasmids were selected for this analysis.

Each defence system encoded on a linear *Streptomyces* replicon (plasmid or chromosome) was then attributed a relative position, ranging from 0 to 1, by normalizing its start position by the length of the replicon it is encoded on. Similarly, each of the BGCs detected by antiSMASH on a linear plasmid or chromosome was assigned a relative position by normalizing their start position by the size of the replicon that encoded them.

## Results

### Distribution of defense systems in actinobacteria

To characterize the anti-phage arsenal of Actinobacteria, we used DefenseFinder [19] to detect all known anti-phage systems encoded in the genomes of the RefSeq database [27] which contains 22 803 fully sequenced genomes (with 2253 actinobacterial genomes belonging to 790 species). In this dataset, not all actinobacterial clades are equally represented. Notably, *Mycobacterium tuberculosis* alone represents 267 genomes. On the other hand, three genomes or fewer are available for 725 of the 790 species of our dataset. In the 2253 actinobacterial genomes, we detected a total of 161 234 defence systems, among which 13 833 were found in Actinobacteria.

Our analysis reveals that Actinobacteria encode on average 6.14 defence systems per genome (Fig. 1a) and dedicate 0.59 % of their genomes to anti-phage defence. Although these numbers are slightly lower than the average in bacterial genomes (7.10 systems per genome on average, representing 0.65 % of the genome), the most important variation does not appear to be between bacterial phyla, but rather between individuals of a given phylum (Fig. S1A). Notably, 36 actinobacterial genomes encode no known defence systems, while the maximum number of defence systems in one actinobacterial genome is 21 (* Olsenella* sp. and *Actinoalloteichus fjordicus*). Out of the 36 genomes with no detected defence systems, ten belong to species known to have an intracellular lifestyle (* Mycobacterium avium *, *Mycobacterium intracellulare, Mycobacterium leprae, Mycobacterium lepromatosis*). The same observation has been made in the past for non-Actinobacteria genomes, suggesting that bacterial lifestyle or bacterial phylogeny might influence the anti-phage arsenal of Actinobacteria [19].

Anti-phage defence systems can be grouped into different types and subtypes, depending on their mechanism and genomic architecture [31, 32]. To characterize the distribution of different types of defence systems in Actinobacteria, we computed their frequencies in this phylum. (Fig. 1b, row ‘All’). Like previously described in other bacterial phyla [19, 33], we observed important heterogeneity in the frequencies of defence systems. While three systems (RM, CRISPR-Cas and Wadjet) are found in more than 40 % of actinobacterial genomes, 63 systems are encoded in less than 5 % of them (Fig. 1b). Interestingly, while the frequency of RM and CRISPR-Cas systems in Actinobacteria (respectively 0.89 and 0.43) is very close to what has previously been reported in all bacteria [19, 33], the Wadjet systems are a lot more abundant in Actinobacteria than in other bacteria (respectively 42 % of Actinobacteria and 7 % of non-Actinobacteria encode at least one Wadjet System, Fig. 1c) [2, 34].

Since the frequency of certain systems (e.g*.* Wadjet) differs in Actinobacteria compared to other bacteria, we then aimed to systematically compare the abundance of the different types of defence systems in Actinobacteria versus in other bacteria. We observed that among the 131 systems that can be detected by DefenseFinder in all prokaryotes, the vast majority are either fully absent (46 systems) from Actinobacteria or are rarer than in other bacteria (42 systems) (Fig. 1d, e).

On the other hand, a minority of systems (14 systems) are more abundant in Actinobacteria compared with other bacteria (Fig. 1d, e). The five systems with the highest enrichment score are systems that are rare in other bacterial phyla and one of them (gp29-gp30, a predicted (p)ppGpp synthetase) is only detected in Actinobacteria (Figs 1c and S1C). As a comparison, more than half of the systems encoded by Proteobacteria are more abundant in this phylum than in other bacteria (Figs 1d and S1B). The molecular mechanisms of the systems enriched in Actinobacteria appear diverse, encompassing notably a Toxin-Antitoxin system (DarTG), a nucleic acid sensing and degrading systems (Wadjet) and several systems of unknown mechanisms (Borvo, Azaca, Tiamat, Uzume …).

Overall, our results suggest that the anti-phage arsenal of Actinobacteria is diverse and currently characterized by a few abundant systems and many rare ones, with important heterogeneity between individuals. The distribution of defence systems appears to be different in Actinobacteria compared to non-Actinobacteria, and some defence systems even appear to be phylum specific.

**Fig. 2.**
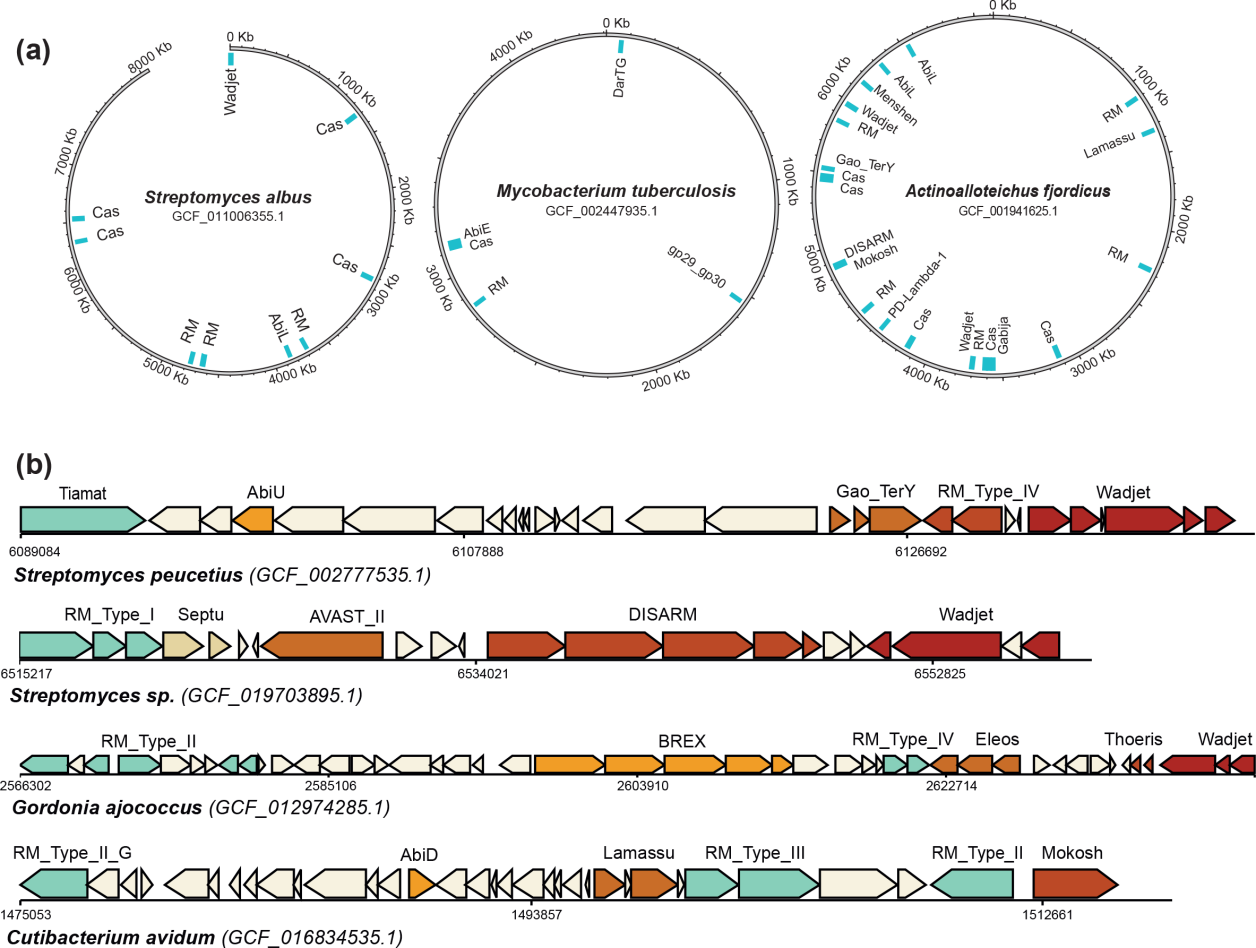
Representatives of the diversity of actinobacterial anti-phage arsenal. (**a.)** Schematic chromosome maps representing the antiphage arsenals of three Actinobacteria of particular interest: *Actinoalloteichus fjordicus*, one of the Actinobacteria encoding the more defence systems (21 systems); *Mycobacterium tuberculosis *, a major human pathogen; *Streptomyces albus *, an actinobacterial model organism. (**b.)** Examples of clusters of defence systems found on actinobacterial chromosomes.

To better illustrate the diversity of actinobacterial anti-phage arsenals, we represented some examples as chromosome maps in Fig. 2(a). Certain Actinobacteria encode clusters of defence systems, with up to six distinct defence systems separated by fewer than 20 proteins. This observation suggests that the tendency of defence systems to colocalize together on the bacterial chromosomes in defence islands [35] is also verified in Actinobacteria. Some examples of defence islands are displayed in Fig. 2(b).

### Phylogeny reveals both shared and different systems within members of actinobacterial genera

To assert how the phylogeny of Actinobacteria impacts the anti-phage arsenal they encode at a lower taxonomic level, we first examined the mean number of defence systems encoded in a genome depending on the genus (Fig. 1a). We observed important variations in the number of defence systems encoded between individuals of the same genus, while no major differences could be established between genera of Actinobacteria. When looking this time at the frequency of different systems depending on the actinobacterial genera, we noticed important disparities. For instance, PD-T4-3 and Eleos are extremely enriched in the *Rhodococcus* genus, while CRISPR-Cas systems are completely absent from the genomes of *Brevibacterium* and *Arthrobacter* of the RefSeq database (respectively 25 and 45 genomes) (Fig. 1b).

**Fig. 3.**
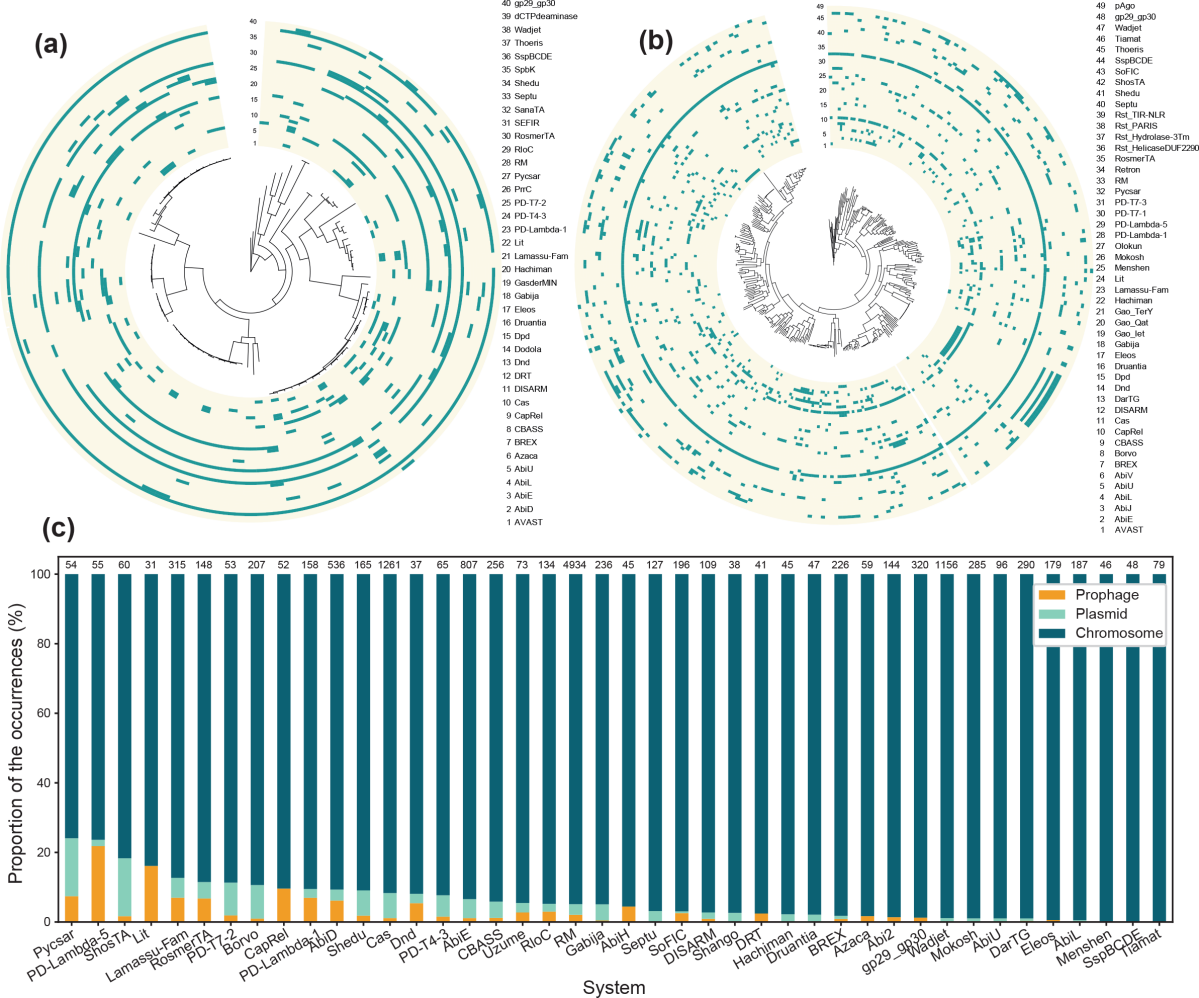
Evidences of Horizontal Gene Transfer in actinobacterial defence systems (a) and (b): Presence (blue) or absence (light yellow) of different types of defence systems in the genomes mapped on the phylogenetic trees (generated using PanACoTA based on the core genome) of two major actinobacterial genera, respectively *Rhodococcus* (a.) and *Streptomyces* (b.). Both trees are rooted on outgroups to the genus (c). Relative contribution (in percentage of the occurrences) of different types of genetic elements to different types of defence systems. Numbers above each bar indicate the total number of occurrences of each type of systems.

We then sought to characterize the link between phylogeny and defence systems distribution at the species level. To do so, we built for each of the major genera of Actinobacteria a phylogenetic tree (Figs 3a, b and S2). As expected from Fig. 1(b), some systems are very abundant while others are very rare in a given genus. The most widespread systems in a given genus are often conserved and spread evenly throughout its branches, rather than constrained to only part of the genus (Fig. 3a, b and S2). For instance, in the case of *Rhodoccus* (97 genomes accounting for 17 species), 73 % of the genomes encode a Wadjet and a RM system and either a Eleos or a PD-T4-3 system (Fig. 3a). Similarly, in the case of *Streptomyces* (344 genomes representing 141 species), 92 % of the genomes encode at least one RM system and 70 % of them encode at least two RM systems (Fig. 3b). On the other hand, certain systems are very rare and unevenly distributed between members of a given genus. For instance, in the *Streptomyces* genus, 28 systems are encoded by less than 5 % of the genomes and are disseminated evenly throughout the tree, while in *Rhodococcus,* 22 systems are found in less than 5 % of the genomes (Fig. 3a, b). To determine whether these rare systems were scattered throughout the tree or confined to specific subclades, we computed for each of *Rhodococcus* and *Streptomyces* systems a measure of the phylogenetic signal with Pagel’s λ (see Methods). For most of the systems encoded by less than 5 % of *Streptomyces* and *Rhodococcus* genomes (respectively 23 and 17 systems), we observed no significant phylogenetic signal (*p* value>0.95), meaning that pairs of closely related strains were no more similar to each other than two randomly selected strains. This suggests that most rare systems in these genera have a patchy distribution.

Overall, the different genera of Actinobacteria seem to often encode a combination of systems that are very widespread within its members and of very rare systems with a patchy distribution. Both the types of shared and variable defence systems vary depending on the genus.

### MGEs contribute to the anti-phage arsenal of actinobacteria

Mobile Genetic Elements (MGEs), such as plasmids, integrative and conjugative elements and phages are known to carry defence systems and contribute to the fast exchange rate of defence systems in bacteria [3, 4, 36–39]. Because the contribution of MGEs to the anti-phage arsenal of bacteria has rarely been characterized outside *Proteobacteria *, we tried to determine this phenomenon in Actinobacteria. To do so, we restricted our analysis to two types of MGEs, plasmids, and prophages. Out of the 13 833 defence systems detected in Actinobacteria by DefenseFinder, 3.4 % are encoded on a plasmid (469). By detecting prophages encoded in the genomes of our dataset using the Virsorter software [21], we found that 2.0 % (279) of the defence systems detected are encoded within a prophage.

Since we observed a heterogeneous distribution of defence systems within Actinobacteria, we then aimed at determining whether some systems are more frequently encoded by MGEs than others. To do so, we calculated for each type of defence system the proportion of the occurrences of the system in a phage, in a plasmid or simply in the chromosome (Fig. 3c). We observed that the proportion of systems encoded on an MGE varies depending on the type of system considered. Some systems are almost always chromosomal (e.g. like Tiamat, or Ssp), while MGEs are important contributors to some other systems. Among the systems that are often encoded by MGEs, some tend to be plasmid-encoded, like ShosTA, while others tend to be prophage-encoded, like PD-Lambda-5 and Lit. The two systems that are the most frequently found on prophages were both discovered in *E. coli* prophages (e14 prophage for the Lit system [40], and P2-like prophage for PD-Lambda-5 [3]), suggesting that the propensity of certain systems to be encoded by prophages might be conserved across bacterial phyla.

Because we had previously observed that the distribution of defence systems is highly variable within Actinobacteria genera, we wondered if the propensity to be MGE-encoded of a given system is linked to its abundance in a given bacterial clade. For each of the major genera of Actinobacteria, we looked at the proportion of occurrences of each type of system that is encoded on an MGE (plasmid or prophage), depending on the proportion of genomes that carry this type of system in the genus (Fig. S3A). We observed that the systems encoded in more than 50 % of the genomes of a given genus were rarely encoded on phages and plasmids.

A lower GC percentage compared to the rest of the genome is a known marker of HGT in bacteria. To explore the link between HGT and the abundance of a system in Actinobacteria, we computed a GC score for each anti-phage gene by normalizing its GC percentage by the GC percentage of the replicon it is encoded on. Thus, a GC score below one means that the gene is enriched in AT compared to the rest of the replicon. We then looked at the average GC ratio of each type of system depending on the proportion of genomes that carry this type of system in a given genus (Fig. S3B) We observed that in all genera studied, the systems with a GC ratio below 0.8 were generally rare systems. Defence systems are known to be among the class of genes with the fastest rate of gain and loss, largely due to events of HGT [41]. On the other hand, variations of GC content have been proposed as a possible indicator of HGT events [42, 43]. Thus, our observations suggest that the rarest systems in a given genus are also the ones that are the most frequently exchanged through HGT, at least partly due to MGEs.

Therefore, it appears that MGEs can be associated with defence systems in Actinobacteria, as observed for other bacterial phyla and notably Proteobacteria. This association probably supports HGT of defence systems between bacteria. MGEs do not contribute equally to all types of defence systems. It also seems that the contribution of MGE to anti-phage arsenals varies depending on the genus, which does not seem linked to differences in the number of MGEs encoded by genomes of the genus (Figs S4A and S4B).

### Comparison of the spatial distribution of defence systems and BGCs reveals conserved trends between two types of gene clusters involved in biological interactions

Actinobacteria are known to carry numerous Biosynthetic Gene Clusters (BGCs), which produce a striking diversity of secondary metabolites [6, 44]. Many of these metabolites are bioactive molecules involved in biological interactions [45], for instance with other bacteria or with fungi. It was recently discovered that several secondary metabolites produced by Actinobacteria had a strong anti-phage activity, a phenomenon that was termed anti-phage chemical defence [17, 18]. This discovery highlights a parallel between BGCs and anti-phage systems in Actinobacteria. Indeed, both are locally adaptive systems that can be used in biological interactions. Because of this parallel, we sought to compare BGCs and defence systems to see if they present similar patterns.

To do so, we used antiSMASH v.6 [20], to systematically detect BGCs in the Actinobacteria genomes of the RefSeq Database. We found that on average, an Actinobacteria genome encodes 17.4 BGCs. Contrary to what we observed with anti-phage systems, the number of BGCs per genome is highly variable depending on the genus (Fig. S5A). As expected from the literature [44], the *Nocardia* and *Streptomyces* genera are much more BGC-rich than other genera, with on average respectively 45 and 46 BGCs per genome. Like for defence systems, we also observe an important heterogeneity in the frequency of different types of BGCs detected by antiSMASH depending on the genus (Fig. S5B). Additionally, we found that BGCs were often plasmid-encoded, and a few seemed to be encoded in prophages (Fig. S5C). These observations are in line with the multiple reports of the importance of HGT in BGCs evolutionary dynamics in Actinobacteria [46, 47], similarly to defence systems.

Not only are certain actinobacterial genera characterized by a rich secondary metabolism, but some, and in particular *Streptomyces,* also have linear chromosomes. *Streptomyces* linear chromosomes are spatially compartmentalized, with a relatively conserved central region and more divergent chromosomal arms. Several studies show that the arms of *Streptomyces* chromosomes are enriched in BGCs [13, 48, 49], but the position of defence systems on these chromosomes has not been characterized. To investigate the consequences of the linearity and compartmentalization of the *Streptomyces* chromosome on anti-phage defence and compare it to what has been observed for BGCs, we selected the chromosomes of all fully sequenced *Streptomyces* genomes available (344 genomes of 141 species). The length of these chromosomes was highly variable (ranging from 1.9 Mbp to 12.3 Mbp with a median of 8.1 Mbp). A recent study on more than a hundred *Streptomyces* chromosomes shows that both the length of the chromosomal arms and of the core region correlate with genome size [50]. Thus, we used a normalized position to systematically characterize the spatial distribution of defence systems and compare it with BGCs.

**Fig. 4.**
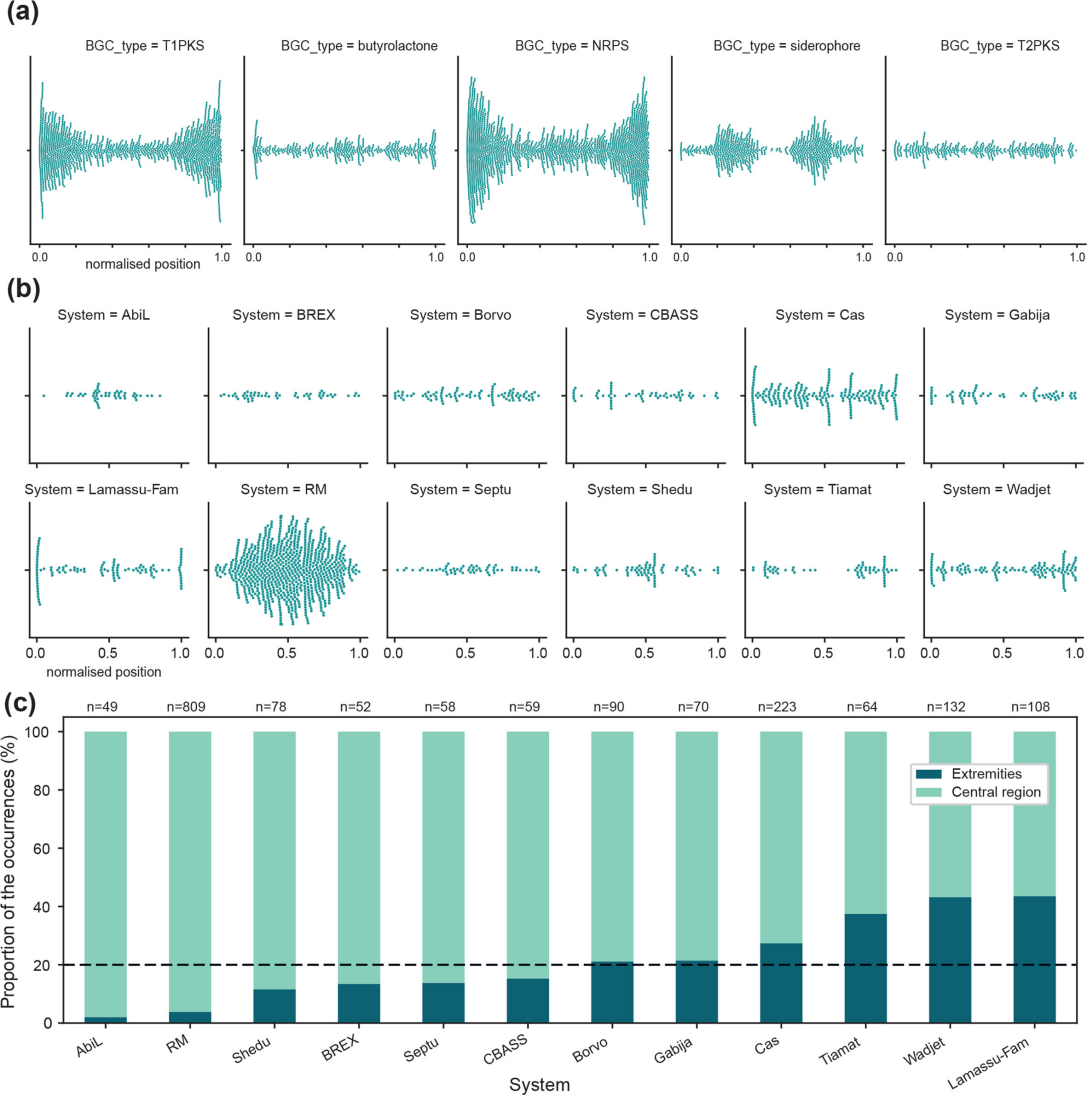
Patterns of spatial distribution of defence systems along *Streptomyces* chromosomes reveals parallels with BGCs. (**a.)** Archetypal examples of the distribution of the normalized position of BGCs along *Streptomyces* linear chromosomes. (**b.)** Distribution of the normalized position of defence systems on *Streptomyces* linear chromosomes. (**c**.) Proportion of each types of defence systems that are encoded in the extremities (first or last 10 %) of the chromosome, versus the ones encoded in the middle (80 %) of the chromosome. The dotted black line indicates the proportion of the chromosome represented by the extremities as defined here, i.e. 20 %. Above each bar is indicated the total number of occurrences of each type of systems in *Streptomyces* linear chromosomes. In (b.) and (c.), only systems with more than 45 occurrences are represented.

As a baseline to characterize the distribution of genes involved in biological interactions, we first mapped the distribution of BGCs along *Streptomyces* chromosomes. As expected from the literature, we observed that certain BGC types (e.g. Type 1 PKS, Type 3 PKS, NRPS…) are strongly enriched towards the extremities of the chromosome (Fig. S6A and S6B). However, we also observe that certain BGCs do not appear to be specifically encoded in the chromosomal arms but are homogeneously distributed along the chromosome (T2PKS for instance). Mapping this time the distribution of defence systems, we also observed specific patterns of spatial distribution that echoed the ones observed for BGCs (Fig. 4b). On one hand, some systems appear to be mostly encoded toward the centre of the chromosome, with the most striking example being RM systems. On the other hand, some systems, such as Lamassu and Wadjet systems, are mostly encoded towards the extremities of the chromosome. As it was previously reported that the length of the arms correlates with the length of the chromosome in *Streptomyces* [50], we calculated the proportion of the occurrence of each defence system within these arms, represented here as the first or last 10 % of the chromosome (Fig. 4c). Less than 4 % of the RM systems are encoded in the chromosomal arms, while this proportion reaches more than 43 % for Lamassu and Wadjet systems. This observation suggests at least two types of spatial organization. We also noticed that Lamassu systems are particularly enriched at the very ends of the chromosome, as almost a third (31 %) of Lamassu systems are found in the first or last 1 % of the chromosome (1 % of the chromosome representing on average 83 kb in this case) (Fig. S7A). Strikingly, Lamassu systems encoded in the first or last 1 % of the chromosome are evenly distributed around the *Streptomyces* phylogenetic tree (Fig. S7B). *Streptomyces* often encode linear plasmids that share their general structural organization with linear chromosomes [12]. The Lamassu systems encoded on one of the 120 *Streptomyces* plasmids annotated as linear on RefSeq also tended to be encoded near the extremities of linear plasmids (Fig. S7C).

Overall, anti-phage defence presents unique spatial distribution patterns in *Streptomyces *, which are linked to the unique genomic organization of these bacteria. These patterns echo the ones observed for BGCs, suggesting that they could be characteristic not of anti-phage defence, but rather of genes involved in biological interactions.

## Discussion

### Database bias

As is often the case in genomic studies, the genome database used in this study is biassed towards certain bacterial clades which are more frequently sequenced. To verify whether this database bias could affect our results, we re-generated our main figures using a reduced set of genomes selected to remove redundancy (see Methods). We did not observe any major changes in the trends reported between our complete set of genomes and this reduced set (Figs. S8–S10).

### Discovery bias: more systems to discover in Actinobacteria and their MGE

In this work, we propose a comprehensive view of the anti-phage arsenal of Actinobacteria. We first observe that Actinobacteria encode many systems. Strikingly, approximately a third of the systems that have been described to date were not detected at all in Actinobacteria. Additionally, among systems encoded by Actinobacteria, most were rarer in this phylum than in other bacteria. One possible explanation for the absence or reduced abundance of most known systems in Actinobacteria could be that they encode systems that are different from commonly characterized bacteria and that have not yet been discovered. Supporting this hypothesis, both the bioinformatic and experimental discovery methods of anti-phage systems are strongly biassed toward certain phylogenetic groups, and notably the Proteobacteria phylum [2–4]. Out of the 131 systems detected by DefenseFinder, only three systems were either from or tested in Actinobacteria (Ssp [51], gp29-gp30[52], and a subtype of BREX [53] named Pgl [54]), against 99 systems from or tested in Proteobacteria. Coincidentally, most defence systems are enriched in Proteobacteria, while only a few are enriched in Actinobacteria. This observation reinforces the idea of a discovery bias, favouring notably *E. coli* and closely related organisms.

Efforts to systematically discover prophage-encoded systems are also strongly biassed toward Proteobacteria [4, 36]. Indeed, we found that the six defence systems that were previously reported as the most prophage-encoded in all prokaryotic genomes (above 60 % of the total number of systems) [19] were almost exclusively found in Proteobacteria (99 % of the occurrences of these six systems). A handful of systems were recently discovered in *Mycobacterium* phages [52, 55–57], which are by far the most characterized and studied actinobacterial phages [58]. Only one of these systems, gp29-gp30, is currently detected by DefenseFinder, and it is exclusively found in Actinobacteria. This supports the idea that novel phylum-specific anti-phage systems can be discovered in bacteriophages infecting Actinobacteria.

Recently, studies focused on Actinobacteria have uncovered novel anti-phage strategies, including transient loss of the cell wall [59] and production of anti-phage compounds [17, 18]. Our results suggest that many more anti-phage systems might be discovered in Actinobacteria and their MGEs.

### Comparison between BGCs and defence systems reveals both shared and distinct characteristics

Because BGCs and anti-phage systems are both conditionally adaptive gene clusters that are often involved in biologic conflicts, we compared these two types of systems in Actinobacteria. We found that both are abundant and widespread in this phylum. Like defence systems, BGCs can be carried by MGEs (Fig. S5C), which probably participates in their HGT.

In the linear chromosomes of the genus *Streptomyces,* we observe that both defence systems and BGCs have two types of distribution. Because *Streptomyces* chromosomes are known to be spatially compartmentalized between a core and a variable region, we initially expected these distributions to be linked to the abundance of the systems (either BGCs or anti-phage). However, the propensity of the relatively abundant Wadjet and Lamassu systems to be encoded in the extremities of the chromosomes seems to contradict this idea. Other hypotheses could explain the observed patterns of distribution along *Streptomyces* chromosomes. Notably, and since the level of gene expression is known to vary depending on the position on the chromosome, the spatial distribution of defence systems and BGCs could reflect different types of regulations [49, 60, 61]. The different patterns of spatial distribution could also be caused by different types of ecological roles or evolutionary dynamics.

### *Streptomyces* linear chromosomes as a model to explore molecular mechanisms of defence systems

The spatial distribution of anti-phage systems on *Streptomyces* linear chromosomes could also provide hints on their mechanisms. Strikingly, the two systems that are the most frequently found at the extremities of the chromosomes (Lamassu and Wadjet) both encompass SMC domain, have anti-plasmid activity, and have been proposed to sense the structure of plasmid DNA [34, 62, 63]. This suggests that the spatial distribution of defence systems might have links with their molecular mechanisms.

Additionally, Lamassus seem to be encoded at the very extremities of the arms. Streptomyces are known to encompass Terminal Inverted Repeat sequences (TIRs) flanking the chromosomal arms at each extremity of the chromosome and of the linear plasmids [12, 64]. The size of these regions varies both intra- and interspecifically, with sizes ranging from less than 1 kb to up to 1 Mb for *S. coelicolor* A3(2) [64, 65]. It is plausible that some of the Lamassus systems that we found to be encoded at the very extremities are part of the TIR region of the chromosome or plasmid. The conservation of Lamassu systems in a region known for its variability is intriguing. Among the many possible hypotheses, it is possible that the chromosomal position of Lamassu systems is linked to their biological function. Exploring patterns of defence systems spatial organization more in depth might provide insights on the function of anti-phage defence systems that remain elusive.

Overall, our results highlight the importance of studying defence systems outside of commonly studied organisms (e.g*. B. subtilis* and *E. coli *), not only to discover novel anti-phage systems but also to characterize the ones that are currently known, both from a mechanistic and ecological point of view.

## Supplementary Data

Supplementary material 1

Supplementary material 2

Supplementary material 3

Supplementary material 4

Supplementary material 5
